# Sensitization Lectures for Reducing Weight Bias in Undergraduate Medical Students

**DOI:** 10.7759/cureus.56431

**Published:** 2024-03-19

**Authors:** Indu Saxena, Rohit Swaroop, Apurva Kumar, Arun K Gupta, Shweta Kumari, Manoj Kumar

**Affiliations:** 1 Biochemistry, All India Institute of Medical Sciences, Gorakhpur, IND; 2 Medicine, All India Institute of Medical Sciences, Gorakhpur, IND; 3 Physiology, Maharshi Vashishtha Autonomous State Medical College, Rampur, IND

**Keywords:** obesity sensitization, obesity destigmatization, obesity bias, obesity, medical students

## Abstract

Introduction: Discrimination exists in one form or another in every society, usually against those who are weaker, in fewer numbers, or different from the rest. Most physicians are empathetic towards their patients but can either not keep an eye on their subordinates or lack the power to act against such employees. Persons experiencing discrimination in healthcare centers may try to avoid or postpone future visits, resulting in delayed diagnosis and treatment of ailments. Obesity bias present in society has crept into healthcare centers and intimidates persons with obesity who are seeking medical aid. Implicit and explicit obesity bias has been recorded in healthcare students.

Methods: Data from 102 undergraduate medical students (23 female) who completed this study was analyzed. Implicit bias (tested online using the Implicit Association Test) and explicit bias (measured using four types of tool kits) were measured before and after conducting an obesity sensitization program (OSP) comprising four lectures on the causes and consequences of obesity and obesity discrimination and its consequences.

Results: The change in implicit bias was not significant. However, a significant reduction was noted in the four different types of tools for explicit bias after conducting the OSP.

Conclusion: OSP helped medical students identify obesity bias and reduce explicit bias. Sensitization lectures conducted in medical colleges and schools can help reduce such discrimination in healthcare centers.

## Introduction

An ideal society should be free from all forms of discrimination, but no nation or society in the world today can claim to be absolutely free from this vice. Holding negative beliefs, attitudes, and assumptions about people being overweight or obese is considered as obesity bias. Victims of discrimination report depression, loss of interest, anxiety, eating disorders, or even feelings of guilt [1]. Compromised performance, development of inferiority complex, dislike for others, self-withdrawal, and isolation may be seen in some victims of discrimination.

Obesity, unfortunately, is considered a stigma that can be openly discriminated against in societies throughout the world [2]. Many people associate obesity with negative stereotypes like lazy, self-indulgent, unmotivated, incompetent, not nice, less intelligent, and untidy [3]. These ideas are strengthened by the belief that people with obesity are responsible for their condition and deserve what they get from society. Anti-obese attitude is present in children as young as three years of age [4]. Clinically overweight children report lower self-esteem than normal-weight children, a condition promoted by weight-related discrimination shown by peers, educators, and, in some cases, the family [5]. Discrimination continues into the college years, where students with obesity have less chances of being selected for admission, especially if they are also female, compared to normal-weight persons with equal academic performance. Obesity discrimination in workplaces results in fewer chances of employment and promotion and higher chances of termination for persons with obesity compared to healthy-weight individuals with identical qualifications [6].

Healthcare centers should be free from discriminatory attitudes, but unfortunately, bias based on race, religion, caste, gender, socioeconomic status, and color is frequently encountered [7]. Many physicians hold their obese patients responsible for their condition, while many are unaware of their own prejudices [3,8]. Patients with obesity may dread hospital visits due to the anti-obese behavior of healthcare providers: the assumption that the patient is unaware of his/her weight problem, an assumption that every health problem in such a person is due to the extra weight, assumption of non-compliance in the overweight, shorter counseling time, and reluctance to perform surgical procedures on such patients. This hesitation to visit a healthcare center is further aggravated if the patients have not been able to reduce their weight as per the physician's advice [9].

Obesity prevalence is increasing in India. The prevalence of obesity based on body mass index and abdominal obesity was 13.85% and 57.71%, respectively, in 2019-21 [10]. Since obesity is associated with various medical conditions [11], physicians can expect more encounters with such patients in their practice. Overt expression of bias is relatively rare in healthcare as socially aware physicians and nurses attempt to be impartial in patient care. However, even the consciously egalitarian healthcare workers may have negatively biased attitudes. The bias and negative vibes experienced by such patients may compel them to delay or postpone hospital visits until the condition becomes serious and becomes a greater financial burden in terms of absence from work, cost of treatment, etc.

Reducing weight bias in healthcare requires recognizing its existence, followed by sensitization of staff. Weight bias can be unconscious or implicit bias, often based on learned associations between particular qualities and social groups. A person may not be aware of their implicit bias and its impact. Weight bias can also be conscious or explicit bias, usually characterized by negative behavior (exclusion, harassment, etc.). Both implicit and explicit bias has been reported in healthcare students [12].

This study was conducted on undergraduate medical students in India to determine the effect of sensitization lectures on implicit and explicit obesity bias.

## Materials and methods

This study was conducted on undergraduate medical student volunteers in the first and second years after obtaining permission from the Institute's Ethical Committee. A total of 350 students were enrolled in the first and second years of the two colleges, of whom 250 were considered potential candidates for this study, as the rest were busy with their exams at the time of the study. Details are summarized in (Table 1). The entire OSP (comprising of 4 lectures) was attended by 173 persons, including some students who were underage or absent on the day of recruitment. Data from only 102 participant students was complete and therefore used for final analysis. Written informed consent was obtained from each student before their participation.

**Table 1:** Details of students available and participating in the study

Details of students available and participating in the study	Number
Total students enrolled in the first and second years of the two colleges	350
Students unable to participate due to their exams	100
Students below 18 years old on the day of commencement of study	53
Students absent on the day and time of enrolment in the study	47
Total number of students enrolled	150
Students absent from at least one out of 4 lectures of the Obesity Sensitization Program	15
Students who incompletely filled tool-kits	32
Students unable to participate in the Implicit Association Test (IAT)	1
Total number of participants	102

The experiment was conducted in three steps: a pretest measurement of bias (implicit or unexpressed bias and explicit or overt bias), a sensitization process, and a post-test repeat measurement of bias.

Pretest measurement of implicit and explicit bias

Measurement of Implicit Bias

Implicit Association Test (IAT) by Project Implicit, 1998 [13] assesses implicit stereotypes held by subjects, such as associations between particular body shape/weight and stereotypes of persons with those qualities. The IAT is conducted online and measures the automatic associations in memory by looking at the subject's reaction time when s/he classifies words. It is used to identify implicit or automatic preferences and biases. Researchers have successfully used the Anti-Fat IAT to measure unconscious anti-obesity bias in physicians and medical students [14].

The students were seated in a lecture hall with their smartphone or laptop with an internet connection and were allowed to complete the implicit association test at a speed convenient to them. After completing the test, the students were asked to screenshot the result. A printout of the results from each student was submitted for evaluation.

Measurement of Explicit Bias

The explicit attitude of a subject was determined using the modules- Attitudes Toward Obese Persons (ATOP) Scale [14], Beliefs About Obese Persons (BAOP) Scale [15], Anti-Fat Attitudes Scale (AFAS) [16], and Fat Phobia Scale (FPS) [17]. The student volunteers were handed the printed copies of the above four modules and were requested to keep their responses anonymous. Filled forms were collected at the end of 45 minutes.

Obesity Sensitization Program (OSP)

This was conducted over 10 days and included four lectures (each of 1 hour duration). Topics included in the lectures are listed below:

Lecture 1: Obesity, techniques used to identify overweight and obesity, and the causes of obesity.

Lecture 2: Metabolically healthy obesity, adiposopathy. Physical, mental, social, and financial consequences of obesity.

Lecture 3: Problems in losing weight, the yo-yo effect of diets. Pharmacotherapy and surgical interventions.

Lecture 4: Types of discrimination faced by persons with obesity in society and healthcare centers, consequences of discrimination on the victim's quality of life, and in seeking medical help for health issues. Need to remove discrimination, non-judgmental attitude, understanding, and empathy, and how to build rapport with such patients.

Post-Test

Tests for implicit and explicit bias were repeated on each subject within 15 days after completion of the sensitization sessions, and pre-and post-test results were compared separately for each test.

Data Analysis

Results of implicit and explicit bias were analyzed separately regarding the percentage of subjects and student t-tests. Pre- and post-test results for each category were compared separately to infer the effect of sensitization lectures.

## Results

Although one hundred and fifty students had initially enrolled in the study, 15 students were absent in at least one lecture of the OSP, 32 students incompletely filled the toolkits, and one student did not participate in the IAT due to technical issues. Thus, results from 102 students have been analyzed. Since only 23 participants were female, the subjects were not categorized into female and male.

The results of the Implicit Association Test (IAT) are summarized in (Table 2). A total of 76 (74.5%) subjects showed an automatic preference for thin people over fat people in the pretest; this number was 70 (68.6%) in the post-test. Twelve (11.8%) subjects in the pretest and 15 (14.7%) subjects in the post-test showed no automatic preference for thin people over fat people. Automatic preference for fat people over thin people was shown by 14 (13.7%) subjects in the pretest and 17 (16.7%) subjects in the post-test.

**Table 2:** Results of Implicit Association Test. Numbers and percentages (in brackets) for pre- and post-test are given against the different categories

Type of Automatic Preference of Respondent	Pre-Test Number (%)	Post-Test Number (%)
Strong automatic preference for thin people over fat people	17 (16.7)	21 (20.6)
Moderate automatic preference for thin people over fat people	23 (22.5)	24 (23.5)
Slight automatic preference for thin people over fat people	36 (35.3)	25 (24.5)
No automatic preference for thin people over fat people	12 (11.8)	15 (14.7)
Slight automatic preference for fat people over thin people	8 (7.8)	9 (8.8)
Moderate automatic preference for fat people over thin people	2 (1.9)	2 (1.9)
Strong automatic preference for fat people over thin people	4 (3.9)	6 (5.9)

Tests for Explicit Bias. Four tool kits were used to assess explicit bias. The results of each were assessed separately.

The ATOP test document has a list of 20 items that focus on stereotypical attitudes about persons with obesity. The respondent indicates the extent of his/her agreement with each separate statement on a Likert scale of -3 (strongly disagree) to +3 (strongly agree). The score is obtained by adding 60 to the points obtained according to the instructions provided with the ATOP tool. Higher scores (>60) indicate a more positive attitude of the respondent towards persons with obesity. In the pretest, only 22.5% of subjects obtained high scores; this number increased to 92.2% in the post-test.

Beliefs About Obese Persons Scale (BAOP) is an eight-item measure that assesses beliefs about the causes and controllability of obesity. The respondents indicate their extent of agreement with statements on a scale of -3 (strongly disagree) to +3 (strongly agree). Points are calculated according to the instructions provided with the BAOP tool, and the score is obtained by adding 24 to the points obtained. Higher numbers (>24) indicate a stronger belief that obesity is not under the person's control. 32.4% of the subjects obtained high scores; this number increased to 89% after attending the Obesity Sensitization Program.

The Anti-Fat Attitude Scale (AFAS) measures the respondent's negative attitude towards obese persons. This scale has 5 items that measure the negative attitude of the respondent towards persons with obesity. The respondent indicates his/ her extent of agreement with the survey statements on a scale of 1 (strongly disagree) to 5 (strongly agree). Item responses are added to obtain the total score. Higher scores (>15) indicate a stronger endorsement of an anti-fat attitude. High scores were obtained by 57.8% of subjects in the pretest; this number decreased to 19.6% after the OSP.

The Fat Phobia Scale (FPS) is a 14-item scale that assesses the stereotypes associated with obesity in the respondent's mind. Fourteen pairs of opposite adjectives used to describe obesity are listed, e.g., fast-slow, weak-strong, shapeless-shapely, etc. The respondents indicate on a Likert scale of 1 to 5 which adjective they feel best describes their feelings or beliefs. The points for each item are added up according to the instructions provided in the tool and then divided by 14. The range of scores is thus 1 to 5. High scores (>2.5) are associated with feelings of greater fat phobia. Pretest scores were high for 52.9% of subjects, and only 29.4% had high scores after the OSP.

Students' t-tests were conducted separately for each test (implicit and explicit), and the results are summarized in (Table 3). No significant difference was observed in the implicit association test (IAT) case. Highly significant differences (p-value < 10-3) were obtained in the case of the tests conducted for explicit bias.

**Table 3:** Summary of results obtained on conducting student’s t-test for results obtained from the tools for determining implicit and explicit bias Abbreviations: IAT: Implicit Association Test; ATOP: Attitude Towards Obese Persons; BAOP: Beliefs About Obese Persons; AFAS: Anti-Fat Attitude Scale

Toolkit Used	IAT		ATOP		BAOP		AFAS		Fat Phobia	
	Pre-Test	Post-Test	Pre-Test	Post-Test	Pre-Test	Post-Test	Pre-Test	Post-Test	Pre-Test	Post-Test
Minimum	1	1	15	23	4	7	6	5	1.6	1.6
Maximum	7	7	89	117	38	39	24	23	4.3	3.7
Mean	2.93	2.97	41.84	72.35	18.92	27.90	15.8	10.61	2.72	2.27
SD	1.46	1.64	19.07	18.51	9.72	6.96	5.91	4.02	0.72	0.44
p-Value		0.857		< 10^-3^		<10^-3^		<10^-3^		<10^-3^

## Discussion

The number of obese people is increasing throughout the world. Since obesity is a risk factor for many diseases, it can be expected that the number of patients with higher than normal BMI approaching healthcare centers will increase in the future. Obesity discrimination in society also enters healthcare centers, creating a frustrating and unwelcome situation for patients with obesity [18,19]. Obesity discrimination in healthcare centers will definitely shoo off such patients, delaying diagnosis and treatment of disease. Persons trained under the principle of 'primum non nocere' (first, do no harm) may harm the patient through their unwelcome attitude and by attributing negative weight-related stereotypes to him/her.

The first step in reducing weight bias is to recognize its existence. Our study shows that many undergraduate medical students have explicit and implicit bias. Implicit bias is formed outside a person's conscious awareness, often based on learned associations between social groups and particular qualities, and is more difficult to remove (Table 2). It significantly affects the person's attitude and behavior and influences decision-making [20,21].

The Obesity sensitization program (OSP) used in this study was intended to make the students aware of the various causes of obesity, to emphasize that body weight may be beyond the person's control, that most patients with obesity are aware of their obese status and have attempted to lose weight, that all ailments in a person with obesity may not be due to their weight, and that negative comments and attitudes about a person's weight cause injury rather than healing. Our study shows that obesity-sensitization lectures can help reduce/remove explicit bias in students (Table 3). Once students are aware of their bias and its consequences, they can modify their behavior to make it more inclusive and empathetic towards patients with obesity.

The limitations of this study are the size of the study and the lack of dramatization in the Obesity Sensitization Program (OSP). A more detailed with a larger number of subjects would further support the role of OSP in removing obesity bias. Since emotional encounters have a greater impact on cognitive and affective domains, including role-play and videos in the OSP would be better.

Obesity is known to be a risk factor for certain diseases [22], and physicians must consider it during diagnosis, treatment, and consultation with patients. This type of discernment is necessary and beneficial for patient care while striving to provide equitable and respectful care to all patients, regardless of their body weight. The governments of all developed and developing countries must realize that the obesity epidemic is here to stay for a reasonably long time. The infrastructure and equipment of the healthcare facilities should be able to accommodate severe obesity. Since weight bias exists in society, healthcare centers, and medical students [23,24], continuous medical education programs should be made compulsory for health providers to reduce anti-fat prejudice. Teachers and senior physicians should address anti-obesity attitudes during the student period so that a more empathetic generation of healthcare providers is conceived. Sensitization lectures can also be conducted in schools as it is easier to influence and modify attitudes in the young.

## Conclusions

Obesity bias is common and is present in undergraduate medical students. This may cause disparities in healthcare. Obesity sensitization programs can be used to identify the existence of obesity bias in students and can also help remove or reduce the bias.
